# Factors influencing the coping abilities in clinic nursing students under public health emergency (COVID-19): a cross-sectional study

**DOI:** 10.1186/s12912-021-00686-0

**Published:** 2021-09-13

**Authors:** Bin Xu, Jian Yu, Suyuan Li, Lu Chen, Zheng Lin

**Affiliations:** 1 Nursing Department, The First Affiliated Hospital with Nanjing Medical University/Jiangsu Provice Hospital, 300 Guangzhou Road, Nanjing, Jiangsu Province China; 2 School of Nursing, Nanjing Medical University, 101 Longmian Avenue, Nanjing, 211166 Jiangsu Province China

**Keywords:** COVID-19, Clinic nursing students, Coping abilities, Psychological and behavioral response

## Abstract

**Background:**

Public health emergencies are serious social problems, threatening people’s lives, causing considerable economic losses, and related to all mankind life and health and safety. Nurses are essential in the fight against the public health emergency, corona virus disease 2019 (COVID-19). clinic nursing students are considered as backup health care providers for licensed nurses, the coping abilities and crisis management of nursing students at present deserve attention all around the world.

**Methods:**

2035 clinic nursing students were invited to participate in mobile phone app-based survey from Feb 6 to 20, 2020. The demographic items, psychological and behavioral responses, and the coping abilities were conducted. Multiple linear regression was used to identify the independent factors to clinic nursing students’ coping abilities under COVID-19.

**Results:**

1992 submitted were valid. Multiple linear regression analysis showed that Confidence to overcome difficulties, Optimism, Active coping, Help seeking and Practice hospital as designated treatment unit were independently associated with the positive coping of clinic nursing students. Fear of COVID-19, Optimism, Avoidance, Help seeking and Severity of epidemic around were independently associated with the negative coping of clinic nursing students.

**Conclusion:**

Under COVID-19, nursing students’ coping level is superior to the Chinese norm, which is also affected by many factors. As the most direct backup resources of professional nurses, the way clinic nursing students respond to public health emergencies and its influencing factors deserve attention.

## Background

The corona virus disease 2019 (COVID-19) is a new highly pathogenic infectious disease caused by the novel beta corona virus [1]. It is reported that more than 45,000 medical staff have gone to the front line to fight against the epidemic in Hubei Province, China, including 30,000 nurses approximately [2]. There is no doubt that nursing staff has played a vital role in the fight and it is not the first time their importance has been highlighted in international emergencies. However, demand for nurses is increasing in all countries and a nine million shortage estimated in 2014 is predicted to decrease by two million by 2030 [3]. clinic nursing students are the most direct reserve of the professional nursing team in the coming years. Research on the psychological state of medical staff during the treatment of patients with COVID-19 showed that as non-front-line care providers, nursing students, like the non-front-line nurses, may suffer more psychological trauma than front-line nurses [4, 5]. In addition, as a sudden public health crisis, the COVID-19 pandemic affect all the college students around the world. Active Minds surveyed 2086 college students regarding the impact of COVID-19 on their mental health and found that 80% of them reported that COVID-19 has negatively impacted their mental health, 1 in 5 of college students say their mental health has significantly worsened under COVID-19 [6].

In China, nursing students are required to do an internship for at least 8 months at the last semester, before they graduate and take the licensure exam to become registered nurses. It prepares nursing students to be able of ‘doing’ as well as ‘knowing’ the clinical principles in practice [7]. Several studies report on internship stress and coping strategies of nursing students during clinical learning. A variety of factors contributing to internship stress including fear of failure, uncertain of ability, theory-practice gap and professional role [8, 9]. Nevertheless,it was reported that clinical nursing students had more active coping styles in China, and the scores of negative coping was lower than the national norm according to the measurement of the Simple Coping Scale [10]. The influencing factors of nursing students choose coping strategies, “Professional preference” and “relevant practical experience” were associated with positive coping and female nursing students are more inclined to positive coping styles [11]. As a sudden public health crisis, the COVID-19 pandemic affect the psychological state of public inevitably. Study showed that the abilities of clinical clinic nursing students to cope with influenza outbreak is not optimistic [12]. Higher levels of stress that may lead to a clear threat to success in a clinical rotation, leading to decrease retention and their dropout from nursing education [13–15], which will have a negative impact on the increase of nursing staff. It’s very important to identify and provide interventions for clinical nursing students with at an early stage. Majority of researches focus on the physical and mental state of the front-line medical staff, but pay less attention to clinical nursing students during the pandemic of COVID-19. This study was conducted to analysis the current status and influencing factors of coping abilities of clinic nursing students under COVID-19 and provides scientific basis for improving the crisis coping abilities of clinic nursing students so as to help them build career confidence.

## Methods

### Settings and participants

Due to the epidemic, this cross-sectional study was conducted in the form of Internet questionnaire via mobile phone app (*https://www.wjx.cn/wjx/design/previewmobile.aspx?activity**=58,349,810&s = 1*) from February 6, 2020 to Feb 20, 2020, in which the purpose and significance of the study were explained. Before the investigation, the researchers contacted the colleges one by one to obtain the consent, and the questionnaire was also distributed through the colleges. Convenience sampling was used to obtain participation among Chinese clinic nursing students from 18 colleges (5 undergraduate and 13 junior colleges), who are doing an internship. They were informed and participation was voluntary. The exclusion criteria include: (1) be absent from clinical practice in the past 3 months; (2) experienced major personal or family events, which may affect their psychological state in the past 6 months, such as traffic accidents or the illness or death of a family member or close friend due to Covid-19, etc. The Ethics Committee of the First Affiliated Hospital of Nanjing Medical University approved the study (approval number: 2019-SR-355).

### Instruments

1. Survey respondents were asked to provide demographic characteristics and information about their proximity and exposure to people with COVID-19 (Table 1).
2. Psychological and Behavioral Responses to Public Health Emergency Questionnaireconsists of three parts, which was compiled by Chinese scholars according to the characteristics of Chinese population [16]. ① Cognitive Response Questionnaire (10 items) including 3 factors: the fear of COVID-19 (4 items, 0–12 points), the confidence to overcome difficulties (2 items, 0–6 points) and the optimism level (4 items, 0–12 points). ② Behavioral Response Questionnaire (10 items) including 3 factors: active coping (5 items, 0–15 points), avoidance (3 items, 0–9 points) and help seeking (2 items, 0–6 points). Responses to items on the Cognitive and Behavioral response questionnaires range from “never” (0 points) to “always” (3 points). Scores for each item within a factor are summed to compute a factor score. ③ Simplified Psychosomatic Symptom Scale (29 items) including 4 factors: somatization (8 items, 8–40 points), anxiety (7 items, 7–35 points), depression (9 items, 9–45 points), hostility (5 items, 5–25 points). Each item is scored using 5-point scale ranging from “strongly disagree” (1 point) to “strongly agree” (5 points). The average score of the factor (the sum of the scores for each item in the factor divided by the total number of items in the factor) ≥ 2 indicates significant positive symptoms. The total score of the scale ranges from 29 to 145. Previous methodological research revealed Cronbach’s a coefficients of 0.686, 0.721 and 0.969 for the Cognitive, Behavioral and Psychosomatic scales, respectively [16]. In our study, the Cronbach’s α are 0.693, 0.733 and 0.948.
3. The Chinese version of the Simplified Coping Style Questionnaire (SCSQ) has 20 items [17], which comprises two dimensions: positive coping (items 1–12) and negative coping (items 13–20). Response options range from “never” (0 points) to “always” (3 points). The score was presented as the average score of positive coping dimension and negative coping dimension separately. The Cronbach’s α coefficients of total scale, positive coping dimension and negative coping dimension reported in previous research were 0.90, 0.89 and 0.78 respectively [18]. In our study, the Cronbach’s α are 0.771, 0.811 and 0.765.

**Table 1:** Demographic characteristics and single factor analysis of coping abilities of clinic nursing students (*N* = 1992)

Demographic characteristics	n(%)	Scores of positive coping dimension (Mean ± SD)	Scores of negative coping dimension (Mean ± SD)	Pairwise comparison
Sex				
Male	142 (7.13)	1.92 ± 0.47**	1.11 ± 0.57	–
Female	1850 (92.87)	2.15 ± 0.59	1.08 ± 0.51	
Education				
Junior colleges	1434 (71.99)	2.15 ± 0.59*	1.09 ± 0.53	–
Undergraduate colleges	558 (28.01)	2.00 ± 0.56	1.05 ± 0.47	
Place of Residence				
City	858 (43.07)	2.06 ± 0.61	1.11 ± 0.52*	There were statistically significant differences between the “city” and “rural” groups
Township	567 (28.46)	2.03 ± 0.57	1.08 ± 0.53	
Rural	567 (28.46)	2.01 ± 0.55	1.04 ± 0.49	
Infected patients around				
With	1557 (78.16)	2.05 ± 0.57*	1.07 ± 0.52*	–
Without	435 (21.84)	1.89 ± 0.62	1.12 ± 0.50	
Severity of epidemic around				
No epidemic	308 (15.46)	2.08 ± 0.60	1.06 ± 0.59*	There were statistically significant differences between the “very serious” and other groups
Not serious	613 (30.77)	2.05 ± 0.55	1.07 ± 0.52	
Not very serious	736 (36.95)	2.01 ± 0.59	1.06 ± 0.50	
Serious	283 (14.21)	2.05 ± 0.59	1.12 ± 0.46	
Very serious	52 (2.61)	1.93 ± 0.66	1.30 ± 0.54	
Hospital grade				
Class III	156 (7.83)	1.99 ± 0.57	1.13 ± 0.52	–
Class II	103 (5.17)	1.97 ± 0.65	1.13 ± 0.56	
Class I	1733 (87.00)	2.05 ± 0.58	1.07 ± 0.51	
Practice hospital as designated treatment unit				
YES	1043 (52.36)	2.28 ± 0.59**	1.09 ± 0.54	There were statistically significant differences between the “yes” and the other groups
NO	512 (25.70)	2.00 ± 0.56	1.06 ± 0.47	
Not clear	437 (21.94)	1.97 ± 0.59	1.08 ± 0.52	
Total	–	2.04 ± 0.58	1.08 ± 0.52	

*:*P*<0.05; **:*P*<0.01

### Statistical analysis

The categorical data is expressed in terms of frequency and percentage (%), and the quantitative data in accordance with the normal distribution is mean ± standard deviation. The data were tested for normality before processing. The independent sample t-test and the one-way analysis of variance (ANOVA) was used to compare the coping styles of nurses with different demographic characteristics. The correlation between continuous variables was tested by Pearson correlation analysis, and that between categorical variables was tested by Spearman correlation analysis. Multiple linear regression (entry method) was used for multivariate analysis due to dummy variables. *P*-value < 0.05 is considered statistically significant. All the analyses were conducted in the statistical software SPSS version 22 (IBM, Armonk, New York).

## Results

### Demographic characteristics and coping abilities of clinic nursing students

Of the 2035 samples invited, 1992 submitted valid surveys, so the effective rate was 97.89%. The coping abilities of clinic nursing students by demographic characteristics is shown in Table 1. The vast majority of the clinic nursing students in the survey were female (92.87%) and nearly three-quarters were junior college nursing students (71.99%). Most of them (87%) studied at first-class hospital and a little more than half of the clinic nursing students (52.36%)are studying in hospitals that are designated for infected patients. 858 (43.07%) students live in cities and 435(21.84%) students found infected patients around them. Sex, Education, Infected patients around and Practice hospital as designated treatment unit are significantly related to the dimension of positive response. Place of Residence, Infected patients around, Severity of epidemic around are significantly related to the dimension of negative response.

### Correlation analysis of the psychological and behavioral responses and coping abilities of the clinic nursing students under COVID-19

According to the clinic nursing students cognitive response, the ‘fear of COVID-19’ factor scored 2.95 ± 1.96 points, the ‘confidence to overcome difficulties’ scored 5.27 ± 1.2 points, and the ‘optimism’ scored 6.59 ± 1.95 points. According to the students’ behavioral response, the score of ‘active coping’ factor was 13.15 ± 2.61, the score of ‘avoidance’ factor was 2.24 ± 1.60, the score of ‘help seeking’ factor was 5.16 ± 2.03. The detection rate of the students who scored ≥2 of different factors was 4.22–8.43% according to the SCSQ. Among them, 84(4.22%) students had ‘somatization’, 117(5.87%) students had ‘anxiety’, 168 (8.43%) students had ‘depression’ and 124 (6.22%) students had ‘hostility’. The correlation analysis results of each factor and coping status is shown in Table 2.

**Table 2:** Correlation analysis of psychological response and coping style of clinic nursing students under emergency

Factors	Mean ± SD/n(%)	Positive coping	Negative coping
**Cognitive response**^△^			
Fear of COVID-19	2.95 ± 1.96	−0.261**	0.173**
Confidence to overcome difficulties	5.27 ± 1.20	0.372**	−0.081
Optimism	6.59 ± 1.95	0.337**	−0.585**
**Behavioral Response Questionnaire**^△^			
Active coping	13.15 ± 2.61	0.373**	0.013
Avoidance	2.24 ± 1.60	−0.232	0.429**
Help seeking	5.16 ± 2.03	0.205**	0.163**
**Simplified Psychosomatic Symptom Scale**^#^			
Somatization *(positive symptoms)*	84 (4.22)	0.220	−0.323
Anxiety *(positive symptoms)*	117 (5.87)	−0.108**	0.283**
Depression *(positive symptoms)*	168 (8.43)	−0.241**	0.192**
Hostility *(positive symptoms)*	124 (6.22)	−0.233**	0.192**

^*△*^:Pearson correlation analysis; #:Spearman correlation analysis; **:*p*<0.01

### Multiple linear regression analysis of influencing factors of coping abilities of clinic nursing students under COVID-19

Multiple linear regression models were fitted with positive and negative coping scores as dependent variables, and statistically significant general data in correlation analysis as independent variables. The assignment of independent variables is shown in Table 3. Due to the existence of dummy variables, the independent variable selection adopts the entry method, with a *p*-value of 0.05 for entering and a p-value of 0.10 for removal. ‘Confidence to overcome difficulties’, ‘optimism’, ‘active coping’, ‘help seeking’, ‘practice hospital is a designated treatment unit for infected patients’ were independently associated with the positive coping of clinic nursing students. ‘Fear of COVID-19’, ‘optimism’, ‘avoidance’, ‘help seeking’, ‘severity of epidemic around’ were independently associated with the negative coping of clinic nursing students. See Tables 4 and 5 (only the independent variables with statistical significance are listed).

**Table 3:** Table of independent variable assignment

Independent variable	Assignment *(Dummy coded)*
Sex	Male = 1; Female = 2
Education	Junior college = 1; Undergraduate = 2
Place of residence	Urban *(Z1 = 0, Z2 = 0)*, Township *(Z1 = 1, Z2 = 0),* Rural *(Z1 = 0, Z2 = 1)*
Infected patients around	With = 1, Without = 2
Severity of epidemic around	No epidemic *(Z1 = 0, Z2 = 0, Z3 = 0, Z4 = 0)*, less serious *(Z1 = 1, Z2 = 0, Z3 = 0, Z4 = 0)*; medium serious *(Z1 = 0, Z2 = 1, Z3 = 0, Z4 = 0)*; more serious *(Z1 = 0, Z2 = 0, Z3 = 1, Z4 = 0)*; very serious *(Z1 = 0, Z2 = 0, Z3 = 0, Z4 = 1)*
Practice hospital as designated treatment uni	Unclear *(Z1 = 0, Z2 = 0)*, Yes *(Z1 = 1, Z2 = 0),* No *(Z1 = 0, Z2 = 1)*
Scores of cognitive response and behavioral response factors	Bring in the original scores
Simplify psychosomatic symptoms	Negative = 1; Positive = 2

**Table 4:** Multiple linear regression analysis of the influencing factors of the positive coping of clinic nursing students

Item	B	SE	β	t	P
Constant	0.799	0.142		5.634	< 0.001
Confidence to overcome difficulties	0.224	0.012	0.049	2.054	0.040
Optimism	0.159	0.007	0.197	8.499	< 0.001
Active coping	0.149	0.005	0.221	9.173	< 0.001
Help seeking	0.037	0.006	0.227	6.011	< 0.001
Practice hospital as designated treatment unit	0.080	0.03	0.169	2.672	0.008

R = 0.450, R^*2*^ = 0.209, Adjusted R^2^ = 0.204, F = 40.315, *P* < 0.001

**Table 5:** Multiple linear regression analysis on the influencing factors of negative coping of clinic nursing students

Item	B	SE	β	t	P
Constant	0.360	0.087		4.161	< 0.001
fear of COVID-19	0.033	0.006	0.125	5.403	< 0.001
optimism	− 0.028	0.006	− 0.205	4.729	< 0.001
avoidance	0.051	0.007	0.157	6.811	< 0.001
help seeking	0.021	0.006	0.082	3.625	< 0.001
Severity of epidemic around	0.182	0.076	0.356	2.392	0.017

R = 0.475, R^2^ = 0.226, Adjusted R^2^ = 0.221, F = 14.724, *P* < 0.001

## Discussion

### Analysis of coping status of different characteristics of clinic nursing students under public health emergency (COVID-19)

In our study, the positive coping dimension scores were higher than that of Chinese norm(2.04 ± 0.58 VS 1.78 ± 0.52, *p* = 0.00), the negative coping dimension scores was less than the Chinese norm (1.08 ± 0.52 VS 1.59 ± 0.66, p = 0.00, 18], which was consistent with the research results of Lin [19]. Clinic nursing students may be somewhat more medically knowledgeable and thus more able to respond to public health emergencies than the general public. From the perspective of general data, in the dimension of positive response, the score of female students is higher than that of male. This is probably due to the expectation of Sex roles. Female are a protected group, When faced with difficulties, they have less pressure and are more willing to take positive measures to achieve psychological balance in the face of difficulties [20]. The positive response scores of clinic nursing students in the designated hospitals for infected patients are higher than the scores of other hospitals that do not treat COVID-19 infected patients, which may be related to the high attention paid to the epidemic by the designated hospitals, and also the adequate protection and comprehensive skill training. In the dimension of negative coping, the score of clinic nursing students living in cities is higher than that in rural areas, which may be related to the epidemic mainly occurs in cities, and the control measures and propaganda in cities are stronger than those in rural areas. In contrast to the results of positive coping, the clinic nursing students with severe epidemic around and with infected patients around had higher negative coping score. Due to their medical background, in the face of unknown epidemic, clinic nursing students may have acute psychological reactions, such as fear, worry about being infected by the disease. The uncertain state of disaster and worries are the awakening factors of fear and anxiety [21], leading to the higher level of their negative coping. Therefore, nursing educators and managers should take different interventions for clinic nursing students of different Sexs especially for male students. In addition, special attention should also be paid to the clinic nursing students who have infected patients around and live in the area where the epidemic is more serious, so as to relieve the group panic psychology and behavior.

### Influencing factors of coping status of clinic nursing students during COVID-19

Confidence and optimism in overcoming difficulties fall under the category of positive psychology, and studies have shown that people with better psychological responses tend to adopt more positive ways to cope with difficulties [21]. Active coping means taking the initiative to take actions because of a threat to one’s health. After the COVID-19 outbreak, health authorities implemented comprehensive prevention and control measures in a timely manner, and schools took active health education activities to increase clinic nursing students understanding of the epidemic, which made their response to the epidemic more obvious, and the score of active response was bound to rise. The medical level of COVID-19 designated units is relatively high, and the epidemic prevention and control publicity and measures are in place, which enhances the confidence of clinic nursing students and encourages them to adopt a more active coping style. Fear and avoidance of COVID-19, as well as the severity of the COVID-19 outbreak in the place of residence, can lead to negative response by clinic nursing students. It is understanderable and interventions should be taken to reduce the fear of COVID-19 so as to reduce the level of negative coping. Help-seeking entered the models of positive and negative coping, both of which had positive effects. However, the coefficient was higher in the model of positive responses, indicating that the change had a greater impact on positive responses. Therefore, it is suggested that nursing educators and managers should give timely feedback on the help-seeking behaviors of clinic nursing students. Although the correlation between anxiety, depression, hostility and coping style in simplified psychosomatic symptoms is statistically significant, they are not included in the final model, which may be related to the low detection rate of psychosomatic symptoms in the clinic nursing students.

### Strategies to improve coping abilities of clinic nursing students under COVID-19

Establish the emergency management mechanism for clinic nursing students. Relevant administrative departments shall formulate and promulgate perfect rules and regulations of ‘contingency plan for clinic nursing students in case of public health emergencies. The administrators should take the initiative to learn new policies, regulations and measures on public health emergencies in time, and strengthen their communication with not only the frontline nurses but also the clinic nursing students [22]. Only by providing relevant information and medical treatments in time can we guarantee the stability of clinic nursing students under public health emergencies, so as to avoid or reduce the panic psychology and behavior of groups.

It is necessary to increase the course of improving students’ crisis coping ability in nursing degree program. The current nursing education system focuses on the cultivation of theoretical knowledge and clinical practice ability, but lacks the cultivation of crisis coping ability in China. Studies have shown that cognitive training can improve the public’s understanding of the unknown, reduce their uncertainty, and provide self relaxation coping skills can also effectively improve the stress state and relieve the psychological pressure [23]. Therefore, in the face of this sudden unknown epidemic, nursing educators and managers should strengthen the training of clinic nursing students on the knowledge of public health emergencies, and advocate initiative response [24]. Furthermore, for the group with poor coping style should be identified and given positive psychological interventions in time. Colleges and teaching hospitals can set up special psychological intervention teams, and provide convenient psychological support for clinic nursing students through group psychology to help improve their confidence and abilities to actively respond to the epidemic, so as to successfully pass the clinical practice.

### Limitations

The study was a descriptive cross-sectional one, and the causal relationship is not very persuasive. Although some strategies for improving clinic nursing students’ coping abilities have been suggested according the results, the effectiveness is unknown. Next step, We will form a series of multi-dimensional interventions and take targeted measures to intervene the clinic nursing students in our hospital as a designated treatment unit.

## Conclusion

Under COVID-19, nursing students’ coping level is superior to the Chinese norm, which is also affected by many factors. As the most direct backup resources of professional nurses, the way clinic nursing students respond to public health emergencies and its influencing factors deserve attention. Much more, it’s necessary to strengthen students’ ability to deal with emergencies, so that they can actively adjust their psychology and improve their coping ability in the face of public health emergencies. This study was a descriptive cross-sectional one, we need more work on how to improve students’ coping styles in the future.

## Data Availability

The datasets used and/or analysed during the current study are availablefrom the corresponding author on reasonable request.

## Abbreviation

COVID-19: Corona virus disease 2019
